# Crystal structure of RNase H3–substrate complex reveals parallel evolution of RNA/DNA hybrid recognition

**DOI:** 10.1093/nar/gku615

**Published:** 2014-07-12

**Authors:** Małgorzata Figiel, Marcin Nowotny

**Affiliations:** Laboratory of Protein Structure, International Institute of Molecular and Cell Biology, 4 Trojdena Street, 02-109 Warsaw, Poland

## Abstract

RNases H participate in the replication and maintenance of genomic DNA. RNase H1 cleaves the RNA strand of RNA/DNA hybrids, and RNase H2 in addition hydrolyzes the RNA residue of RNA–DNA junctions. RNase H3 is structurally closely related to RNases H2, but its biochemical properties are similar to type 1 enzymes. Its unique N-terminal substrate-binding domain (N-domain) is related to TATA-binding protein. Here, we report the first crystal structure of RNase H3 in complex with its RNA/DNA substrate. Just like RNases H1, type 3 enzyme recognizes the 2′-OH groups of the RNA strand and detects the DNA strand by binding a phosphate group and inducing B-form conformation. Moreover, the N-domain recognizes RNA and DNA in a manner that is highly similar to the hybrid-binding domain of RNases H1. Our structure demonstrates a remarkable example of parallel evolution of the elements used in the specific recognition of RNA and DNA.

## INTRODUCTION

RNases H are small nucleases that specifically hydrolyze RNA moieties in various double-stranded nucleic acids that contain both RNA and DNA (1,2). Two structural classes of RNases H are designated H1 and H2, and the latter is further divided into RNases H2 and H3 (1,2). The canonical substrate of type 1 RNase H is an RNA/DNA hybrid, in which the cleaved strand is composed of RNA, and the other strand is composed of DNA. The minimal substrate for RNase H1 is a DNA duplex with a stretch of four ribonucleotides in one strand (3). RNase H1 is involved in the removal of R-loops from the DNA structures that arise when nascent transcribed mRNA hybridizes with complementary DNA strand in the transcription bubble (4) or are produced when Rad51 promotes RNA–DNA strand exchange (5). RNase H1 is also essential for mitochondrial DNA replication (6), although the exact mechanism remains to be established. RNase H1 is also an indispensable domain of retroviral reverse transcriptases, in which its activity is required at several steps in the conversion of viral genomic RNA into double-stranded DNA in the reverse transcription process.

The mechanism of substrate recognition and cleavage by RNase H1 was elucidated based on a series of structures of bacterial and human enzymes (7,8). The RNA strand of the hybrid is recognized by contacts between the protein and 2′-OH groups of two ribonucleotides on each side of the scissile phosphate, and the DNA strand is recognized through its flexibility. Both bacterial and human enzymes have a pocket that tightly binds the DNA phosphate group located across the minor groove from the scissile phosphate. The interaction with this pocket leads to a deformation of the non-cleaved strand that requires it to adopt a B-form conformation with a narrow minor groove and northern sugar puckers that are only allowed for DNA. The active site of RNases H1 consists of negatively charged carboxylate residues that coordinate two divalent metal ions, preferably magnesium (7,9). Manganese is known to relax the enzyme's specificity, whereas calcium inhibits its activity (9). One of the metal ions (ion B) destabilizes the substrate, and the other ion (ion A) coordinates and activates a water molecule that performs a nucleophilic attack on the scissile phosphate (9,10). In addition to the catalytic domain, some RNases H1 also contain a small ∼50-amino-acid N-terminal hybrid-binding domain (HBD). Its function is to anchor the enzyme on the substrate and allow multiple cleavages in one region of the substrate to enhance the enzyme's processivity (11).

The key feature of RNase H2 is its ability to cleave RNA and DNA junctions in double-stranded nucleic acids before the last RNA residue (3,12). In fact, bacterial enzymes are only able to cleave such substrates, whereas eukaryotic enzymes are additionally capable of cleaving RNA/DNA hybrids in the middle of the RNA sequence (13). RNases H2 efficiently perform cleavage when even a single nucleotide is present, which is important for their *in vivo* activity (12,14). RNase H2 is the only known enzyme that initiates a mutation-free process of single ribonucleotide removal from the DNA (15,16). Such single ribonucleotides are very common and need to be efficiently removed because they lead to instability of the genome (17). RNase H2 has also been shown to play a role in the processing of R-loops at stalled replication forks, complementing the activity of Sgs1 helicase (18).

RNase H2 utilizes a unique mechanism for the specific recognition of RNA–DNA junctions (14). Through a network of tight interactions, it binds the 2′-OH group of the ribonucleotide of the junction. A highly conserved tyrosine forms a stacking interaction with the sugar ring of the second residue of the junction, excluding the presence of 2′-OH and leading to preference for DNA. The RNA–DNA junction is deformed by the enzyme, allowing the phosphate group between RNA and DNA to participate in the coordination of metal ion A. The substrate is thus used to assemble the active site and promote its own catalysis, ensuring the exquisite specificity of RNase H2. Recently, substitutions were made in two amino acids of yeast RNase H2 based on comparisons of the structures of unliganded *B. stearothermophilus* (Bst) RNase H3 with *T. maritima* (Tm) RNase H2–substrate complex. These changes resulted in abolishing its activity on RNA–DNA junctions but not on R-loops, rendering the yeast enzyme similar to an RNase H1 (18).

RNase H3 was only found in some bacteria from taxonomically very divergent groups and a few species of archaea (19). It is very closely related to RNase H2 at both the sequence and structure level and appears to have diverged from RNase H2 very early in evolution (20). However, quite strikingly, its biochemical properties resemble type 1 enzymes, in which it prefers to cleave RNA/DNA hybrids and hydrolyze them in the middle of the RNA sequence (3,12,21,22). The analysis of the distribution of the three types of RNases H among bacterial species showed that most bacterial genomes contain a combination of H1 and H2 or a combination of H2 and H3 (19). The combination of only H1 and H3 has not been found in any of the prokaryotic genomes. Therefore, RNase H3 has been suggested to function as a replacement for RNase H1.

RNases H3 contain an N-terminal domain (N-domain) that structurally resembles the TATA-binding protein (TBP) but has a monopartite structure, whereas TBP contains two very similar modules for the symmetric binding of DNA (23). Based on sequence and structural data, TBP and the N-domain have been proposed to have evolved from a common ancestral DNA-binding domain that fused with RNase H2 to produce RNase H3 or formed TBP through gene duplication (20). The RNase H3 N-domain enhances substrate binding (21,22) and is important for the *in vivo* function of the enzyme (22). However, the exact mode of substrate binding, mechanism of specific recognition of RNA/DNA hybrids by N-domain, and its functioning in full-length RNase H3 remain unknown.

Despite the similarity to RNase H2, the type 3 enzyme exhibits the biochemical properties of RNase H1. In order to explain this, we sought to solve a crystal structure of RNase H3 in complex with its RNA/DNA substrate. The structure revealed a remarkable example of parallel evolution of the elements required for discrimination between RNA and DNA in both domains of RNase H3. Our structure completes the family picture of RNases H and suggests a universal set of elements used by enzymes to distinguish RNA from DNA.

## MATERIALS AND METHODS

### Protein preparation and structure solution

A full description of the methods can be found in the Supplementary Information. Briefly, *T. ammonificans* RNase H3 carrying a His-tag and a SUMO-tag was expressed in *E. coli* and purified on a nickel column, followed by tag cleavage and further purification on the nickel column. Crystals of Ta-RNase H3 in complex with a 19-bp RNA/DNA hybrid oligonucleotide were obtained in 0.2 M AmSO_4_, 0.1 M BisTris (pH 5.5), 25% PEG 3350 and 10 mM spermine tetrahydrochloride. The diffraction data for the crystals were collected at the Berliner Elektronenspeicherring-Gesellschaft für Synchrotronstrahlung (BESSY) synchrotron at beamline MX-14.1 on a Pilatus 6M detector at 100K (24). The data sets were processed and scaled using XDS (25). The structure was solved with Phenix Autosol using the single-wavelength anomalous diffraction (SAD) method and the data set collected with KAuCl_4_-soaked crystals. Refinement of the structure was performed with Phenix (26) interspersed with rounds of manual corrections in Coot (27). According to MolProbity analysis, all of the residues reside in the allowed regions of the Ramachandran plot.

### Biochemical assays

The RNase H cleavage assay and electrophoretic mobility shift assay (EMSA) were performed by standard methods using fluorescently labeled substrates. A detailed description can be found in the Supplementary Information.

## RESULTS

### Protein purification and crystallization

Working toward a crystal structure of RNase H3–substrate complex, we purified the enzyme from three different thermophilic bacterial species: *Thermovibrio ammonificans* (Ta), *Thermocrinis albus* (Tha) and *Thermotoga lettingae* (Tl). The activity of the three proteins was characterized (Supplementary Figure S1), and all were less active on a DNA duplex with a single ribonucleotide than on a substrate with a stretch of four ribonucleotides (D_6_-R_4_-D_14_/D—a duplex with one strand comprising six deoxyribonucleotides followed by four ribonucleotides followed by 14 dexoyribonucleotides, and the second strand comprising DNA only). For Ta-RNase H3 an approximately 1000-fold higher amount of protein was needed to achieve similar activity on the single ribonucleotide substrate, confirming the preference of the enzyme for longer stretches of RNA. For the D_6_-R_4_-D_14_/D substrate, the first cleavage occurred between the first and second ribonucleotide and the second cut 5′ of the RNA nucleotide of the RNA–DNA junction. The exact mechanism of the two cuts is not clear at this point but may be determined by sequence preference of the enzyme. It is also possible that the presence of the first nick aids conformational changes of the substrate required for the second cut right before the RNA–DNA junction to occur.

**Figure 1.**
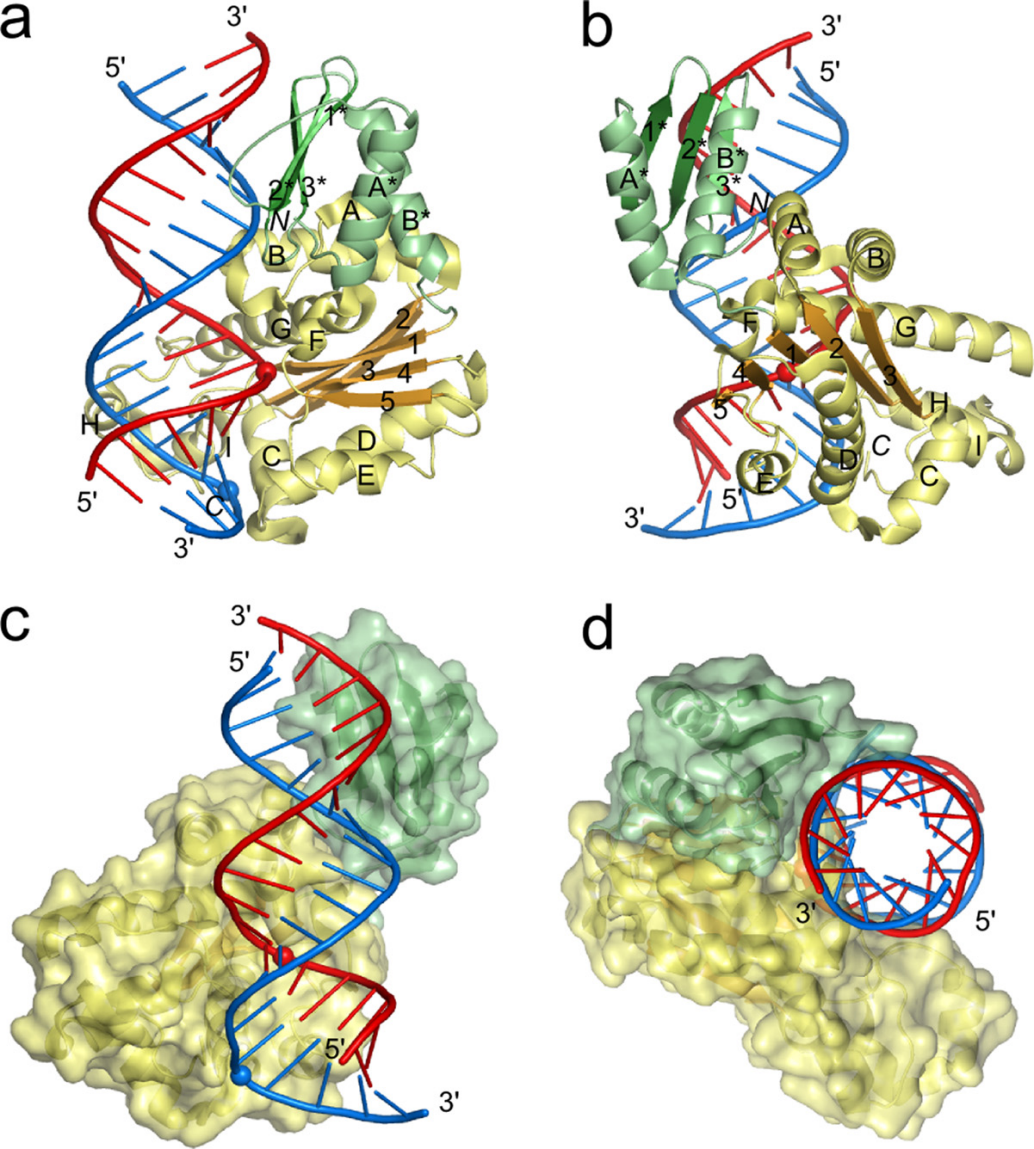
Overall structure of the Ta-RNase H3-substrate complex. (**a**) and (**b**) Cartoon representation of the structure with secondary structure elements labeled with numbers (strands) and letters (helices) independently for each domain (elements of the N-domain are indicated with an asterisk). The N-domain is colored in shades of green, and the catalytic domain is colored in yellow and orange. DNA is shown in blue, and RNA is shown in red. The scissile phosphate and the phosphate that interacts with the phosphate-binding pocket are shown as spheres. The orientation in panel (b) versus panel (a) is related by a 90° rotation around the y-axis. (**c**) and (**d**) Structure of the complex with the protein in surface representation. The orientation in panel (d) versus panel (c) is related by a 90° rotation around the z-axis followed by a 90° rotation around the x-axis.

All three proteins underwent extensive crystallization trials in the presence of various RNA/DNA hybrids. The best crystals were obtained for Ta-RNase H3 in complex with a 19-mer RNA/DNA and diffracted X-rays to 2.1 Å resolution. The structure was solved by single-wavelength anomalous diffraction using crystals soaked with KAuCl_4_. The initial experimental density maps were of poor quality but sufficiently well defined to manually place a homology model of the protein that was subsequently manually rebuilt. The model of RNA/DNA substrate was next added, and the complex structure was refined (Table 1 and Figure 1). Examples of electron density maps can be found in Supplementary Figure S2. Crystal packing does not appear to influence the protein–nucleic acid interactions (Supplementary Information, Supplementary Figure S3).

**Table 1.** Data collection and refinement statistics

	Au soak	Native
**Data collection**		
Space group	*P*2_1_2_1_2_1_	*P*2_1_2_1_2_1_
Cell dimensions		
*a*, *b*, *c* (Å)	59.4, 68.2, 108.8	58.4, 67.7, 107.5
*α*, *β*, *γ* (°)	90, 90, 90	90, 90, 90
Resolution (Å)	50.0–2.2 (2.33–2.2)	50.0–2.1 (2.23–2.1)
*R*_merge_	11.9% (42.3%)	6.3% (49.7%)
*I* / *σ*	15.41 (5.21)	18.06 (2.66)
Completeness (%)	96.3 (78.9)	99.3 (95.7)
Redundancy	7.1 (6.6)	6.2 (4.3)
**Refinement**		
Resolution (Å)		2.1
Number of reflections		25392
*R*_work_/*R*_free_ (%)		18.32/23.3
No. of atoms		3053
Protein		2036
Nucleic acids		788
Ligand		6
Ion		36
Water		187
Average *B*-factors		23.2
Protein		21.4
Nucleic acids		26.2
Ligand		30.4
Ion		41.0
Water		26.1
Root-mean-square deviations		
Bond lengths (Å)		0.007
Bond angles (°)		1.07

Values in parentheses are for highest-resolution shell.

The N-domain of Ta-RNase H3 comprises the first ∼70 residues and contains a three-stranded antiparallel β-sheet that in the sequence is flanked by two α-helices (A* and B*, N-domain elements are denoted with asterisks). In the structure, the helices cover one surface of the sheet (Figure 1a and b). The catalytic domain of ∼200 amino acids adopts the RNase H fold (Figure 1a and b). Its central β-sheet is composed of five strands numbered as they appear in the sequence and arranged 5–4–1–2–3, with strand 2 running antiparallel to the other strands. The A and B helices are inserted between strands 2 and 3 of the central β-sheet and form a unique element of RNases H2 and H3, absent from other enzymes that adopt the RNase H fold (28,29). Groups of three (C, D, E) and two (F, G) helices are located on the sides of the central β-sheet. On top of the β-sheet are two short helices, H and I, that form the C-terminal helical extension of the RNase H fold.

The individual domains of the previously determined structures of Bst and *A. aeolicus* (Aae) RNases H3 are very similar to those in our Ta-RNase H3 structure. For example, for Aae-RNase H3 and Ta-RNase H3, the N-domains can be superimposed with a root-mean-square deviation (rmsd) of 1.1 Å (54 C-α atoms), and the catalytic domains can be superimposed with an rmsd of 1.3 Å (151 C-α atoms). However, the relative position of the two domains is different in each of the three structures, indicating the flexibility of their arrangement (Supplementary Figure S4). In Aae-RNase H3 and Bst-RNase H3 structures, the arrangement of the domains does not allow for their simultaneous binding to the substrate, suggesting that a conformational change that involves restructuring of the linker between the two domains is required.

In Ta-RNase H3, the linker is short and composed of three residues. The domains interact with each other through hydrophobic contacts between helix B* from the N-domain and helix A from the RNase H2/3-specific insertion in the catalytic domain. In the structure, both domains interact with the RNA/DNA substrate, and the protein covers a continuous stretch of 15 bp of the hybrid (Figure 1c and d). As a result of a cloning artifact, the N-terminus of the protein contains two additional residues, Ser and Met. These residues form a very short β-strand located at the interface between the two domains, and the N-terminal serine forms contacts with the DNA backbone. The short strand occupies the same position as the additional N-terminal β-strand observed in the N-domains of the Bst- and Aae-RNase H3 structures (21,22). Therefore, we conclude that the presence of two additional residues should not affect the structure.

### Substrate binding by the catalytic domain

**Figure 2.**
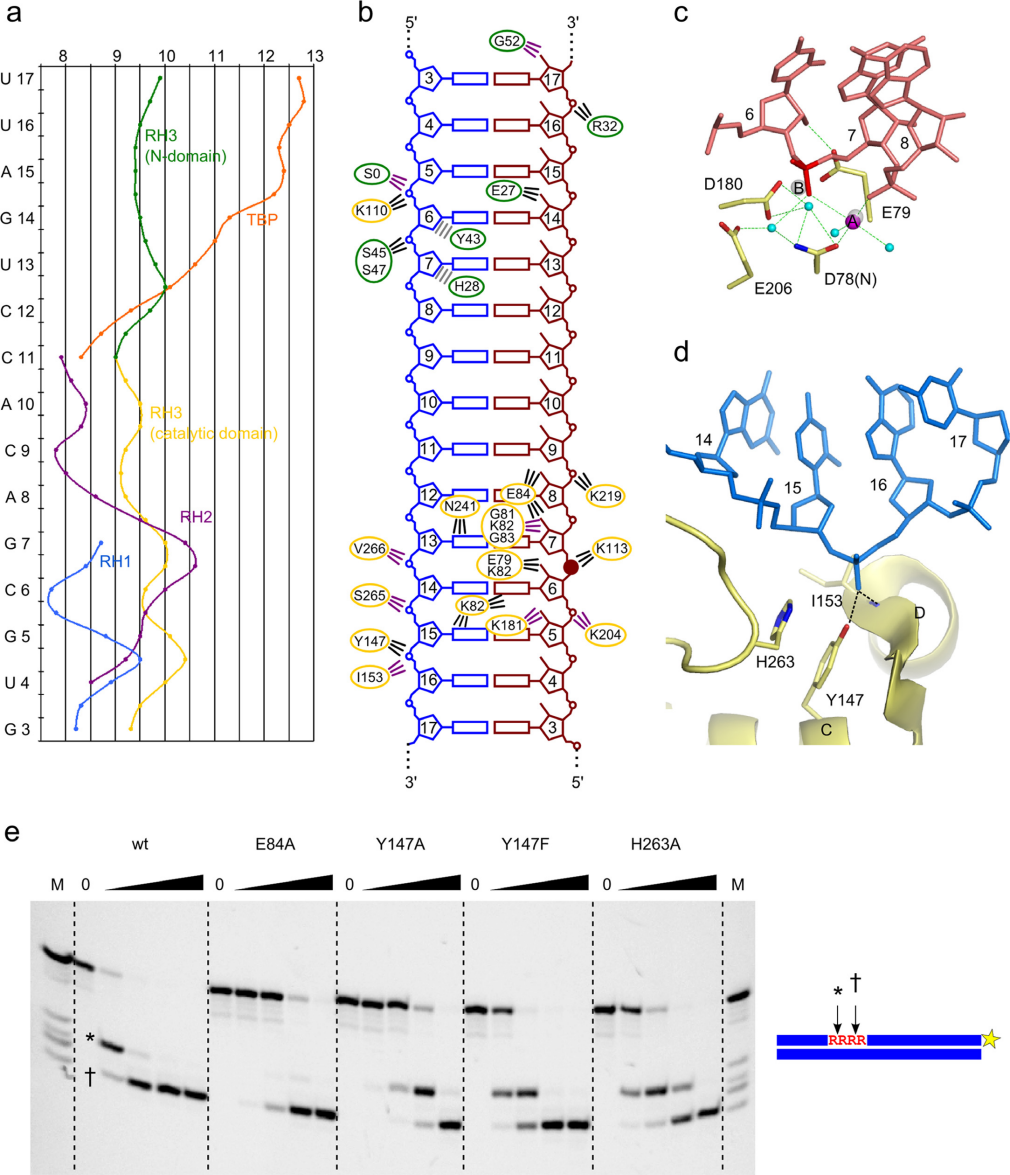
Substrate binding by the catalytic domain. (**a**) Minor groove width by residue. The curves depict the distribution of the minor groove width in the nucleic acids bound by RNase H1 (RH1, blue), RNase H2 (RH2, purple), RNase H3 (RH3 catalytic domain, yellow; RH3 N-domain, green) and TATA-binding protein (TBP, orange). Alignment of the plots was guided by the structural superimposition of protein-structure complexes onto the Ta-RNase H3 structure. (**b**) Schematic representation of protein–nucleic acid contacts. The DNA strand is in blue, and the RNA strand is in red. The ovals are color-coded for the domains (green for N-domain and yellow for catalytic domain). The lines indicate interactions and are color-coded (interactions with the amino acid side chain, black; interactions with peptide backbone, purple; van der Waals contacts, gray). The scissile phosphate is indicated with a solid circle. (**c**) Active site of Ta-RNase H3. Active site residues are shown as sticks and labeled. The fragment of the RNA strand is shown as pink sticks, and the scissile phosphate is indicated in red. Magnesium ion A is shown as a magenta sphere. Water molecules are shown as cyan spheres. The positions of metal ions A and B from superimposed Tm-RNase H2 structure (PDB ID: 3O3G (14)) are shown as gray spheres. (**d**) Phosphate-binding pocket of Ta-RNase H3. Residues involved in interactions with phosphate are shown as sticks and labeled. DNA fragments are shown as blue sticks. (**e**) Cleavage of D_6_-R_4_-D_14_/D substrate (at 500 nM concentration) by Ta-RNase H3 substitution variants. The lane without the enzyme added is indicated by 0. The lanes marked with a triangle contain increasing concentrations of protein (0.5, 5, 50, 500 nM). The reactions were incubated at 37°C for 15 min in the presence of 5 mM MgCl_2_. The cleavage products were analyzed on 20% Tris borate EDTA-urea polyacrylamide gels. The size of the products was estimated by comparisons with the marker indicated as M (products of alkaline hydrolysis of the cleaved strand). The two cleavage sites are indicated with * and †, and the fluorescent label position is indicated with a yellow star.

In the Ta-RNase H3 complex structure, the RNA strand of the hybrid, as expected, adopts an A form-like conformation with C3′-endo sugar puckers. The residues of the DNA strand, with the exception of one nucleotide, adopt the A form conformation with northern sugar puckers. The minor groove has a width between 9 and 10 Å, indicating a conformation intermediate between A and B but closer to the A form (Figure 2a).

The RNA strand is bound at the edge of the central β-sheet of the catalytic domain. In addition to multiple van der Waals interactions, Lys113 and Lys219 bind the phosphate backbone of the RNA (Figure 2b). The first residue is absolutely conserved in other RNases H3 and also type 2 enzymes and participates in the formation of a functional DSK motif that stabilizes the scissile phosphate at the active site (14). Lys219 is conservatively replaced in other RNase H3 sequences (Supplementary Figure S4f). The scissile phosphate is located between ribonucleotides 6 and 7 (Figure 2b). 2′-OH groups of four ribonucleotides, two on each side of the scissile phosphate, interact with the protein, leading to RNA-specific binding: ribonucleotide 5 interacts with the backbone carbonyl of Lys181, ribonucleotide 6 interacts with Glu79, and ribonucleotides 7 and 8 interact with the side chain of Glu84. The last residue is replaced by an aspartate or serine in some RNase H3 sequences (Supplementary Figure S4f). The 2′-OH group of ribonucleotide 7 also interacts with the backbone amide groups of Gly81, Lys82 and Gly83. In RNases H2, an equivalent Gly-Arg-Gly (GRG) motif enables recognition of the ribonucleotide at the RNA/DNA junction (14). In RNases H3, only the glycines are strictly conserved, so the motif can be defined as GXG. Additional contacts are between Lys82 and Asn241 and the base edges of the hybrid's minor groove.

The scissile phosphate is located at the active site of the enzyme composed of four carboxylates: Asp78 (replaced in the crystallized protein with Asn to inactivate the enzyme), Glu79, Asp180 and Glu206 (Figure 2c). The importance of the corresponding residues for activity has been shown for Bst-RNase H3 and Aae-RNase H3 (21,22). One metal ion, refined as Mg^2+^, is observed at the active site, corresponding to metal ion A. It exhibits regular octahedral coordination with six ligands: pro-Sp non-bridging oxygen of the phosphate group of nt 7, pro-Rp oxygen of nt 8, backbone carbonyl of Gly79, side chain of Asn78 and two water molecules (Figure 2c). One of the waters is positioned to perform nucleophilic attack on the scissile phosphate that is conducive to RNA hydrolysis. The metal ion B binding site is occupied by a water molecule. We attribute the lack of the second metal at the active site to D78N substitution, which also alters the conformation of this residue. The binding of metal ion B, however, is well documented in the structures of other RNases H3 (21) and RNase H2 (14) , where it is coordinated by a pro-Sp non-bridging oxygen and 3′-O of the scissile phosphate. In Ta-RNase H3, this ion would also interact with the backbone of Glu79 and side chains of Asp78 and Glu79. The last residue also binds a 2′-OH group of the substrate and therefore couples the RNA recognition with metal ion coordination and active site assembly.

The RNA at the active site is distorted, with backbone torsion angles beta and gamma of the ribonucleotide 8 differing from canonical values by 90° and 120°, respectively. The deformation results from the pushing of ribonucleotide 8 from the regular helix due to the interaction of the RNA with a loop before the C-terminal helical extension. The deformation allows the phosphate of this ribonucleotide to participate in the coordination of metal ion A. Thus, in RNase H3, this deformation plays a critical role in active site assembly as has been shown for RNase H2 (14).

The interactions between the catalytic domain and DNA are less extensive than for the RNA strand. The most notable DNA interaction is formed by the phosphate group of nucleotide 16 located across the minor groove from the scissile phosphate, two base pairs (bp) from it (Figure 2b and d, Supplementary Figure S5). The non-bridging oxygen of this nucleotide forms hydrogen bonds with the amide backbone of Ile153 and side chain hydroxyl group of Tyr147. The latter residue in other RNase H3 sequences is often replaced by glutamine (Supplementary Figure S4f), which could also form an efficient interaction with the phosphate group of the DNA. The aromatic ring of Tyr147 is stabilized by stacking with His263. We consider the element that is composed of these three residues the phosphate-binding pocket, analogously to the element found in RNases H1. It is located at the N-terminus of helix D, and the positive side of the helical dipole further contributes to the binding of the phosphate group. The interaction with the phosphate-binding pocket induces a deformation of the DNA (Figure 2a and d), manifested by large changes in the alpha and gamma backbone torsion angles of nucleotide 16, which differ from canonical values by ∼170° and ∼120°, respectively. Additionally, nt 15 is the only nucleotide in the DNA that adopts the B form-like C2′-endo southern sugar pucker. Because this conformation is not allowed for RNA, the deformation of the non-cleaved strand serves for the detection of DNA. A similar mechanism has been identified for RNA/DNA recognition by RNase H1 (7,8). In addition to the phosphate-binding pocket, highly conserved Phe264 is located in the vicinity of C2′ of the ribose of nt 14. The presence of 2′-OH would lead to a clash with the aromatic ring of the phenylalanine, leading to further discrimination against RNA. Consistent with our structural data, the biochemical studies showed that RNases H3 do not hydrolyze duplexes with RNA as the non-cleaved strand (12). In our structure, Asn154, Arg155, Asn184 and Arg185 interact with a sulfate ion and could also potentially interact with the non-cleaved strand as discussed in the Supplementary Information.

### Mutational verification of residues involved in substrate binding

To verify the importance of the substrate interactions observed in the structure, we prepared variants of Ta-RNase H3 with substitutions in the residues that are involved in nucleic acid binding in the structure (Figure 2e). The wildtype protein and substitution variants were mixed with a fluorescently labeled 24-mer RNA/DNA hybrid, and the reaction products were monitored by polyacrylamide gel electrophoresis (PAGE). The first variant tested was E84A, with substitution of the residue that recognizes two 2′-OH groups of the RNA. Its activity was reduced by two orders of magnitude, confirming the importance of Glu84. We then tested the effect of substitutions in the phosphate-binding pocket. The Y147A and Y147F variants had reduced activity by approximately 100-fold and 10-fold, respectively, demonstrating the importance of the pocket and the hydroxyl group of the tyrosine residue. The H263A variant was also affected (∼10-fold activity reduction), showing that although this residue is not directly involved in substrate binding, it is important for the integrity of the phosphate-binding pocket.

### N-domain

**Figure 3.**
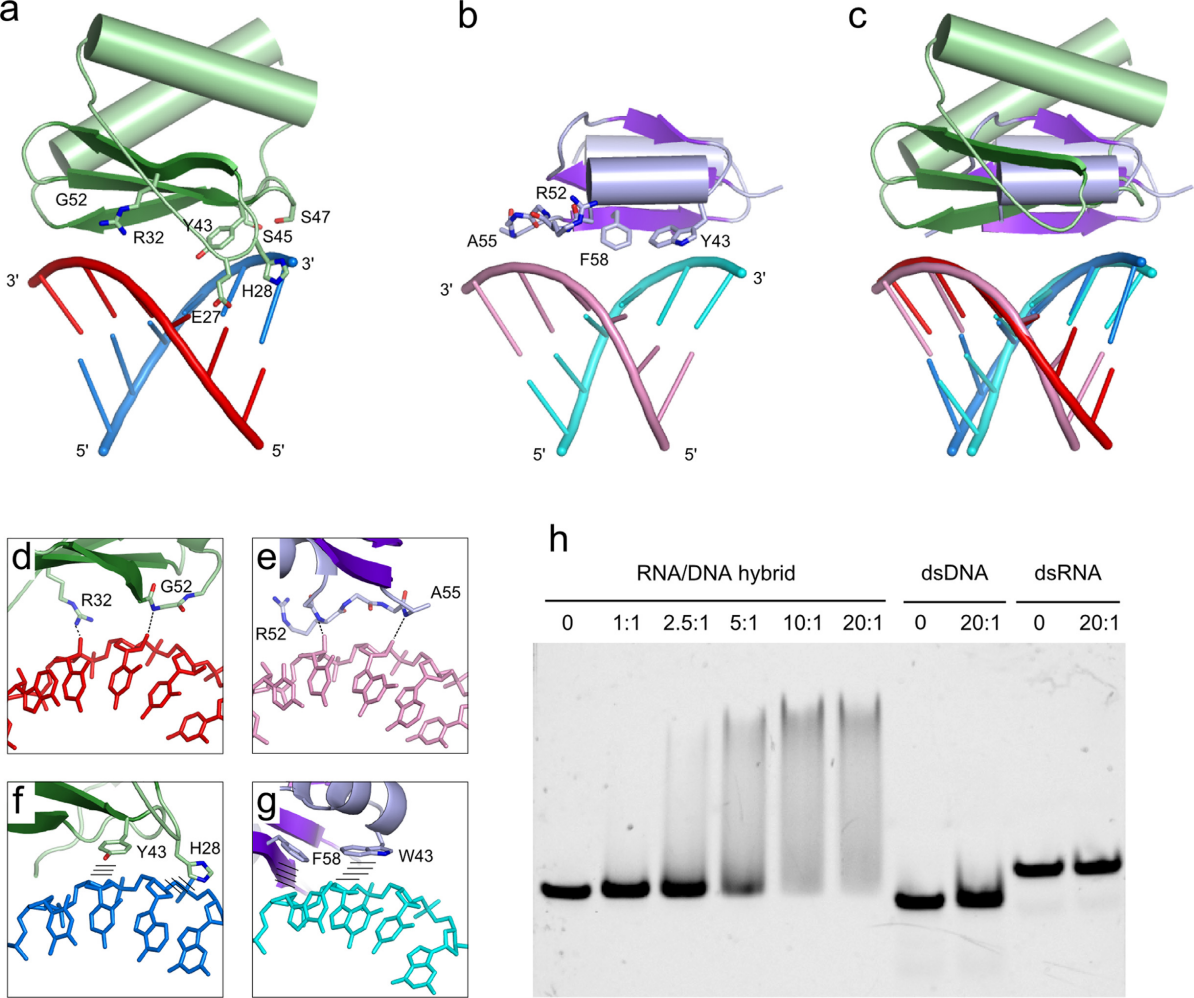
Substrate binding by the N-domain. (**a**) N-domain of RNase H3 bound to an RNA/DNA hybrid. DNA is shown in blue, and RNA is shown in red. Residues that interact with the nucleic acid are shown as sticks and labeled. (**b**) HBD of human RNase H1 domain (PDB ID: 3BSU (11)) bound to an RNA/DNA hybrid. DNA is shown in cyan, and RNA is shown in pink. Residues that interact with the nucleic acid are shown as sticks and labeled. (**c**) Superimposition of N-domain of RNase H3 and HBD of RNase H1 protein–substrate complexes. (**d**) and (**e**) Close-up view of selected interactions that provide recognition of the RNA strand by (d) N-domain and (e) HBD. Interactions are indicated with the dashed lines. (**f**) and (**g**) Close-up view of selected interactions that provide recognition of the DNA strand by (f) N-domain and (g) HBD. Stacking interactions between aromatic side chains and deoxyribose rings are indicated with lines. (**h**) EMSA of nucleic acid binding to the isolated N-domain of Ta-RNase H3. The samples contained equal amounts of fluorescently labeled 24-bp substrates. The protein:substrate molar ratio in the sample is indicated. Control lanes with no protein added are indicated by 0. The samples were resolved on a 10% native TAE polyacrylamide gel.

In our structure, the exposed surface of the N-domain β-sheet binds the hybrid across the minor groove roughly 10 bp downstream from the scissile phosphate (Figures 1, 2b and 3). The contacts with the phosphate backbone of the RNA strand are limited to a single weak interaction mediated by Arg32, but the protein forms RNA-specific contacts with 2′-OH groups, in which the backbone amide of Gly52 interacts with nucleotide 17 and the side chain of Glu27 interacts with nucleotide 14 (Figure 3a and d). Interactions with the backbone of the DNA strand are also few and involve binding of the phosphate of nucleotide 7 by serines 45 and 47. Important are the van der Waals interactions between the sugar rings of deoxyribonucleotides 6 and 7 and aromatic rings of Tyr43 and His28, respectively (Figure 3a and f). If RNA were present in those two positions, then the 2′-OH groups in the ribose rings would clash with the aromatic rings of Tyr and His, and thus these interactions are selective for the DNA strand. Therefore, the structure implies that by combining the interactions with the 2′-OH and stacking of aromatic rings with deoxyriboses of the DNA, a relatively small N-domain can achieve specific binding of an RNA/DNA hybrid.

The substrate specificity of the N-domain was tested by the EMSA (Figure 3h). In agreement with the prediction made based on our structure, the isolated N-domain only bound RNA/DNA hybrid (albeit weakly) and did not interact with dsDNA or dsRNA.

## DISCUSSION

Here, we describe the structure of RNase H3 from *T. ammonificans* complexed with an RNA/DNA hybrid. We provided structural information on substrate binding by the N-domain, which is only the second example of a small domain that is capable of specific RNA/DNA hybrid recognition. The N-domain is a structural relative of the TBP. A detailed comparison of the N-domain and TBP can be found in the Supplementary Information (Supplementary Figure S6). Some residues in the N-domain have been previously predicted to interact with the substrate based on comparisons with TBP–DNA complex structures. Their importance was verified in site-directed mutagenesis studies (30) and is in excellent agreement with our structural data. Alanine substitution of equivalents of the DNA-interacting Tyr43 residue significantly reduced the Mg^2+^-dependent nucleolytic activity in Bst-RNase H3 and Aae-RNase H3 (22,30). In Bst-RNase H3, the substitution of the equivalent of Ser45, which interacts with the phosphate of the DNA in our structure, led to the moderate inhibition of activity (30). In Bst enzyme, the side chain of Gln54 occupies similar position in space to Ta-RNase H3 RNA-binding Gly52. The substitution of Bst Gln54 led to a 40-fold reduction of activity (30), suggesting that the side chain of Gln mediates 2′-OH binding. All of these described substitutions also led to a reduction of substrate affinity.

**Figure 4.**
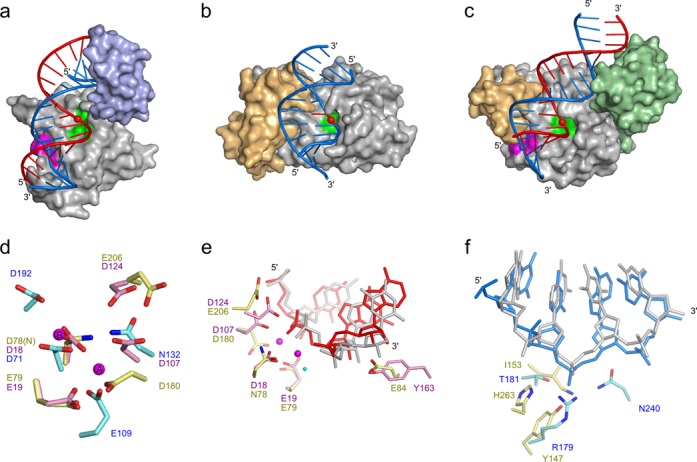
Comparison of RNase H3 with type 1 and 2 RNases H. (**a**)-(**c**) Surface representations of protein–substrate complexes. (a) Model of the full-length RNase H1 (PDB IDs: 2QK9 (catalytic domain) (8) and 3BSU (HBD) (11)), (b) RNase H2 (PDB ID: 3O3G (14)) and (c) Ta-RNase H3. The model of RNase H1 was prepared by combining the structures of isolated catalytic domain and HBD both in complex with RNA/DNA. The flexible linker joining the two domains allows for a range of distances between them when they both bind the substrate. For the model, the shortest distance not leading to any clashes between the domains was arbitrarily chosen. The nucleic acid substrates are shown in cartoon representation and color-coded (blue for DNA, red for RNA). Scissile phosphates in the RNA and phosphates bound in the phosphate-binding pockets are shown as spheres. Active sites are marked in green, and phosphate-binding pockets are marked in magenta. Additional domains are shown in light-blue (HBD), light-orange (C-terminal helical extension), and light-green (N-domain). (**d**) Superimposition of the active sites of RNase H1 (cyan), RNase H2 (pink) and RNase H3 (yellow). Magnesium ions from Tm-RNase H2 structure (PDB ID: 3O3G) are shown as magenta spheres. (**e**) Superimposition of the active sites of RNase H2 (pink, nucleic acid in gray) and RNase H3 (yellow, RNA in red). The structures were superimposed using the central β-sheet. The magnesium ions (magenta) and attacking water molecule (cyan) are shown as spheres and positioned as determined for Tm-RNase H2. (**f**) Superimposition of phosphate-binding pockets of RNase H1 (cyan, DNA in gray) and RNase H3 (yellow, DNA in blue). The structures were superimposed using the DNA fragments.

The catalytic domain of RNase H3 bears a strong resemblance to RNases H2. Ta-RNase H3 can be superimposed on Tm RNase H2 with an rmsd of 2.35 Å over 170 C-α atoms (Figure 4b and c). This conservation also applies to the architecture of the active site (Figure 4d). The positioning of the carboxylates and binding of metal ion A are nearly identical, which is probably also the case for metal ion B. Both enzymes also possess a loop termed DSK in RNases H2, which is essential for the enzyme's activity and stabilizes the scissile phosphate. Importantly, the deformation of the substrate 1 nt from the scissile phosphate is observed in RNase H3, which is also a characteristic feature of RNase H2 (Figure 4e).

Despite the high conservation of the structure and active site, RNase H2 and RNase H3 differ in substrate preference. Bacterial type 2 enzymes cleave only RNA–DNA junctions, whereas type 3 enzymes prefer regular RNA/DNA hybrids, similar to RNase H1 (Supplementary Figure S1) (12). Our structure suggests a basis for this difference. RNase H3 forms contacts with the 2′-OH groups of four consecutive RNA residues, two on each side of the scissile phosphate. This pattern is remarkably similar to RNase H1. Many of these interactions are not observed in RNase H2. For example, the backbone carbonyl of Lys109 (equivalent to Ta-RNase H3 Lys181) cannot interact with the substrate because of an alteration of the protein backbone conformation. Moreover, in RNase H2, the position of the side chain of Glu84, which binds two 2′-OH groups in Ta-RNase H3, is occupied by a tyrosine that in fact precludes RNA binding by forming a stacking interaction with the sugar ring of the DNA residue of the RNA–DNA junction (14). This tyrosine is one of the most important determinants of substrate specificity of type 2 enzymes. Its replacement with glutamine with additional P45D substitution converts RNase H2 enzyme to RNase H1-like activity and preference for RNA/DNA hybrids (18).

Another important difference between type 2 and type 3 enzymes is that bacterial type 2 enzymes can cleave substrates with RNA as the non-cleaved strand, whereas H3 enzymes require DNA as the complementary strand (12). Our structure explains this by showing that a phosphate group of the DNA strand that is located across the minor groove from the scissile phosphate is bound tightly by the protein, leading to deformation of the non-cleaved strand, which is only allowed for DNA. Quite strikingly, interactions of the DNA with the phosphate-binding pocket in type 1 and type 3 enzymes lead to nearly identical deformations of the nucleic acid (Figure 4f).

The feature shared by RNase H1, H2 and H3 is the location of the deformed phosphate close to the N-terminus of the first helix of the canonical RNase H fold (helix D in Ta-RNase H3). The positive pole of the helical dipole of this helix may stabilize the deformed phosphate in all three types of RNases H. There are, however, important differences between these enzymes. In the structures of Tm-RNase H2 the binding of the deformed phosphate does not involve any protein side chains (14), while in RNase H1 (7,8) and RNase H3 amino acid side chains interact with the phosphate. The requirement of RNases H1 and H3 for DNA as the non-cleaved strand of the substrate (12) is likely mediated by these side chain interactions. Because in RNase H1 and H3 very different amino acid residues located in different regions of the protein sequence contribute to phosphate binding, we postulate that these interactions in the two enzymes evolved independently.

The domain organization of RNase H3 and RNase H1 is similar. Both enzymes contain, in addition to the catalytic domain, a small N-terminal domain that is responsible for substrate binding (HBD in type 1 enzymes and the N-domain in RNases H3) (Figure 4a and c). Although the topologies of the two domains are very different, the mechanism of RNA/DNA binding bears remarkable similarities (Figure 3a–c). In HBD, two elements are used for RNA/DNA recognition: (i) two adjacent RNA nucleotides form RNA-specific interactions between their 2′-OH groups and amide groups of the protein backbone (Figure 3b and e), and (ii) the deoxyribose rings of two DNA nucleotides form DNA-specific stacking interactions with aromatic rings of two protein side chains (11) (Figure 3b and g). Functional equivalents of these two elements found in the N-domain are the interactions with 2′-OHs mediated by Glu27 and Gly52 and stacking interactions between DNA and His28 and Tyr43 (Figure 3d and f). Quite strikingly, the two elements have a very similar spatial arrangement in HBD and the N-domain, spanning the shortest distance across the minor groove. Together with the emergence of the phosphate-binding pocket, the mode of substrate recognition by small N-terminal domains of type 1 and type 3 RNases H constitutes a remarkable example of parallel evolution.

Our structure thus provides two examples of the apparently independent emergence of protein modules used for the specific discrimination of RNA from DNA. The fact that these elements arose independently suggests that they may constitute a universal set of elements used by proteins when discrimination between RNA and DNA is required. As expected, the contacts with 2′-OH groups of RNA are the main element for RNA recognition. However, DNA detection is more complex. Nucleic acid deformation to B form-like conformation is one strategy utilized by both RNase H1 and H3. Another recurring element for DNA recognition is stacking interactions between deoxyribonucleotide sugar rings and aromatic amino acid side chains. Several such interactions can be found in RNases H. For example, it is present at the active site of RNase H2 and used for specific RNA–DNA junction recognition (14). In RNases H1, it is found in HBD and in a helical element called basic protrusion. In human RNase H1, this element forms a tight channel that accommodates the DNA strand of the RNA/DNA substrate. A tryptophan side chain lines this channel, forming a stacking interaction with a ribose ring and selecting for the DNA (8). Outside of RNases H, a prominent example of stacking interactions are the so-called ‘steric gates’ of DNA polymerases, which preclude the binding of incoming rNTP at the active site (31). By substituting the aromatic ring of this steric gate, HIV-1 reverse transcriptase can be converted to an RNA polymerase (32).

In summary, our structure completes the picture of the mechanism of RNases H. RNase H3 represents a notable example of conversion of the substrate specificity from RNA–DNA junction recognition to RNA/DNA hybrid cleavage. This conversion seems to have involved a remarkable case of parallel evolution of the substrate recognition elements employed by RNases H. It also points to the universal principles of RNA and DNA recognition and discrimination by proteins. The recurring elements include the detection of RNA by contacts with 2′-OH groups and the detection of DNA by deformation to B form-like conformation and stacking of deoxyribose with aromatic amino acid side chains.

## ACCESSION NUMBERS

The atomic coordinates of the Ta-RNase H3–substrate complex structure have been deposited in the Protein Data Bank under accession number 4PY5.

## SUPPLEMENTARY DATA

Supplementary Data are available at NAR Online, including [33].

SUPPLEMENTARY DATA
