# Supplementary material for: Crystal structure of RNase H3–substrate complex reveals parallel evolution of RNA/DNA hybrid recognition
Source: Nucleic Acids Res. 2014 Jul 12;42(14):9285–94. doi: 10.1093/nar/gku615 (PMC4132731; doi:10.1093/nar/gku615)
Supplement: SUPPLEMENTARY DATA [file supp_42_14_9285__index.html]

Crystal structure of RNase H3–substrate complex reveals parallel evolution of RNA/DNA hybrid recognition — Crystal structure of RNase H3–substrate complex reveals parallel evolution of RNA/DNA hybrid recognition — SUPPLEMENTARY DATA 

# Crystal structure of RNase H3–substrate complex reveals parallel evolution of RNA/DNA hybrid recognition

## SUPPLEMENTARY DATA

**Files in this Data Supplement:**

- SUPPLEMENTARY DATA
