# Supplementary material for: Crystal structure of RNase H3–substrate complex reveals parallel evolution of RNA/DNA hybrid recognition
Source: Nucleic Acids Res. 2014 Jul 12;42(14):9285–94. doi: 10.1093/nar/gku615 (PMC4132731; doi:10.1093/nar/gku615)
Supplement: SUPPLEMENTARY DATA [file supp_gku615_nar-00846-h-2014-File006.pdf]

Crystal structure of RNase H3-substrate complex reveals parallel evolution of RNA/DNA hybrid recognition

Małgorzata Figiel and Marcin Nowotny

SUPPLEMENTARY INFORMATION

MATERIALS AND METHODS

Protein expression and purification

Synthetic genes that encode *Thermovibrio ammonificans* (Ta), *Thermocrinis albus* (Tha), and *Thermotoga lettingae* (Tl) RNase H3 were purchased from Epoch Life Science and subcloned into a pET28 expression vector that carries an N-terminal His-tag and SUMO-tag that is removable by SENP protease. BamHI and XhoI restriction sites were used for cloning, which results in two N-terminal residues after SENP cleavage: Serine 0 and initiator Methionine 1. Mutagenesis of the constructs was performed using the QuikChange protocol (Stratagene) or inside-out polymerase chain reaction (PCR). For expression of the isolated Ta-RNase H3 N-domain (residues 1-70) a stop codon was introduced into the expression construct for the full length protein.

For expression, the vectors were transformed into *Escherichia coli* BL21 Star cells (Ta-RNase H3 and Tha-RNase H3) or RIL cells (Tl-RNase H3). Protein expression was induced overnight with 0.4 mM isopropyl 1-thio-β-D-galactopyranoside at 30°C. Bacterial cells were next suspended in 40 mM NaH_2PO_4 (pH 7.0), 100 mM NaCl, and 5% glycerol with the addition of a mixture of protease inhibitors and incubated on ice in the presence of 1 mg/ml lysozyme. After sonication, the cleared lysate was applied to a HisTrap column (GE Healthcare) equilibrated with 10 mM imidazole, 40 mM NaH_2PO_4, 0.5 M NaCl, and 5% glycerol. After a wash step with 60 mM imidazole, the protein was eluted with 300 mM imidazole. Eluted fraction was dialyzed overnight against 10 mM imidazole, 40 mM NaH_2PO_4, 0.5 M NaCl, and 5% glycerol in the presence of SENP protease. The sample was then reapplied to the HisTrap column in the same conditions. Cleaved protein did not bind to the resin and was collected in the flow-through fraction. Pure protein was concentrated to 25-56 mg/ml and stored in 20 mM Tris (pH 8.0), 100 mM NaCl, 5% glycerol, 0.5 mM EDTA, and 1 mM DTT.

Ta-RNase H3 N-domain expression was performed in *Escherichia coli* BL21star cells and induced overnight with 0.4 mM isopropyl 1-thio-β-D-galactopyranoside at 16°C. Two-step purification on a HisTrap column was performed as described for the full length protein. The collected fraction was concentrated and applied to a Superdex 75 column equilibrated with 20 mM Tris (pH 8.0), 100 mM NaCl, 5% glycerol, 0.5 mM EDTA, and 1 mM DTT. Fractions containing the pure protein were pooled and concentrated to 21.5 mg/ml.

Crystallization

HPLC-purified oligonucleotides were ordered from Metabion (Martinsried, Germany) and Future Synthesis (Poznan, Poland). The 19 bp hybrid was created by annealing the RNA strand (5'-GAGUGCGACACCUGAUUCC-3') with the complementary DNA strand (5'-GGAATCAGGTGTCGCACTC-3'). The D78N variant of Ta-RNase H3 was mixed with the hybrid in a 1:1.2 protein:substrate molar ratio. The final concentration of the protein used for crystallization was 4 mg/ml. The complexes were mixed with the reservoir solution at equal volume and crystallized using the sitting drop vapor diffusion method at 18°C. The native Ta-RNase H3 crystals were obtained with 0.2 M $AmSO_4$, 0.1 M BisTris (pH 5.5), 25% PEG 3350, and 10 mM spermine tetrahydrochloride. For data collection, the crystals were transferred to a cryoprotecting solution that was identical to the well solution but contained 35% PEG 3350 and flash frozen in liquid nitrogen. The Ta-RNase H3-Au crystals were obtained with 0.2 M $AmSO_4$, 0.1 M BisTris (pH 6.0), 26% PEG 3350, and 0.1 M sodium citrate tribasic and soaked with 10 mM $KAuCl_4$ in a cryoprotecting solution that contained 29% PEG 3350.

Diffraction data collection and structure determination

The diffraction data for all of the crystals were collected at the Berliner Elektronenspeicherring-Gesellschaft für Synchrotronstrahlung (BESSY) synchrotron at beamline MX-14.1 on a Pilatus 6M detector at 100K (24). The data for the native and Au-soaked crystals were collected at wavelengths of 0.91841 Å and 1.04024 Å, respectively. The datasets were processed and scaled using XDS (25). The crystals belonged to the $P2_12_12_1$ space group and contained one protein-substrate complex in the asymmetric unit.

The structure was solved with Phenix Autosol using the SAD method and the dataset collected with Ta-RNase H3-Au crystals. Homology models of the catalytic and N-domains were generated with SwissModeller (http://swissmodel.expasy.org/) using *A. aeolicus* RNase H3 structure (PDB ID: 3VN5) and C-α traces of those models were manually docked into the electron density maps. The nucleic acid model was manually built into the structure in Coot. Refinement of the structure was performed with Phenix (26) interspersed with rounds of manual corrections in Coot (27). After preliminary refinement of the model using the dataset collected for Ta-RNase H3-Au crystals, the structure was further refined against the diffraction data collected for the native Ta-RNase H3 crystals, resulting in better resolution and statistics. Of the reflections, 5% were used to calculate the R_{free}. In the final model, all of the residues reside in the allowed regions of the Ramachandran plot. The structure contains one molecule of glycerol and seven sulfate ions. Structural analyses and figure preparation were performed using PyMol (www.pymol.org/).

Crystal packing interactions are shown in Supplementary Figure 3. They involve stacking of the blunt ends of the hybrids, which nearly form a pseudohelix going through the crystal. Additional protein-protein crystal contacts are between helix H (Lys249) and helix B (Glu129). Very weak contacts between the RNA strand of the hybrid and the symmetry-related protein molecules are

mediated by Arg232 and Lys258. All the observed crystal contacts are far from the substrate interface and therefore do not seem to affect protein-nucleic acid interactions.

RNase H cleavage assays

The substrate specificity and activity of Ta-RNase H3 was determined using 24-mer RNA/DNA substrates labeled with TAMRA at the 3' end of the cleaved strand. The sequences of the nucleotides were the following (ribonucleotides are indicated by small lettering): D_9-R_1-D_{14} (5'-ACAGAGTGCgACACCTGATTCCAT-3'), D_6-R_4-D_{14} (5'- ACAGAGugcgACACCTGATTCCAT-3'), RNA (5'-acagagugcgacaccugauuccau-3'). All were hybridized to DNA (5'-ATGGAATCAGGTGTCGCACTCTGT-3'). The substrate concentration was 500 nM, and the enzyme concentrations were 500, 50, 5, and 0.5 nM. The reactions were performed for 15 min at 37ºC in a buffer that contained 75 mM NaCl, 20 mM Tris (pH 8.0), 5% glycerol, 1 mM DTT, 100 µg/ml BSA, and 5 mM $MgCl_2$. The hydrolysis products were analyzed on 20% Tris borate EDTA-urea polyacrylamide gels and visualized by fluorescence readout.

Electrophoretic mobility shift assay

The substrate specificity of the isolated N-domain of Ta-RNase H3 was determined using 24-mer RNA/DNA hybrid, dsDNA and dsRNA of the same sequence labeled with TAMRA at the 3' end of one of the strands. The substrate concentration was 1 µM and the protein was added in 1:1, 2.5:1, 5:1, 10:1 or 20:1 molar ratio to the substrate. Reactions were incubated at room temperature for 30 min in a buffer that contained 100 mM NaCl, 20 mM Tris (pH 8.0), 5% glycerol, 1 mM DTT, 0.5 mM EDTA and 100 µg/ml BSA. The samples were resolved on a 10% native polyacrylamide gel in 1 × TAE buffer at 4ºC and visualized by fluorescence readout.

SUPPLEMENTARY RESULTS

Comparison of the N-domain and TBP

The main difference between the N-domain and TBP is that the former has a monopartite structure, and the latter is composed of two similar halves (TBP-domains). A single domain of the human TBP can be superimposed on the N-domain of Ta-RNase H3 with an rmsd of 2.7 Å over 30 C-α atoms (Supplementary Fig. 6a). The highest structural similarity is observed for the three β-strands that are common for the two domains (1*, 2*, and 3* in the N-domain), whereas the positions of the two helices present in the fold are less conserved. Because the role of the TBP-domains in the TATA-binding protein is the sequence-specific binding of dsDNA in the promoter region, it establishes both interactions with the DNA backbone (e.g., Arg294 and Ser307) and base-specific contacts (e.g., Phe288 and Phe305; Supplementary Fig. 6b). A few interactions are conserved between Ta-RNase H3 and TBP, including DNA backbone phosphate-binding by Ser45 (Ser307 in human TBP) and a stacking interaction that discriminates against 2'-OH by Tyr43 (Phe305). Despite these similarities, a large difference is observed in terms of nucleic acid conformation. The dsDNA bound by the TBP-

domain retains Watson-Crick base-pairing but is largely unwound, with minor groove width values that oscillate between 11 and 13 Å (Fig. 2a), whereas the hybrid bound by the N-domain preserves the A form-like conformation.

Additional potential interactions with the non-cleaved strand

When structures of Tm-RNase H2 (14) and Ta-RNase H3 are compared, the trajectory of the non-cleaved strand downstream from the phosphate-binding pocket differs. In RNase H3 the DNA strand leaves the protein surface and does not form any further contacts with the protein (Fig 2b, Supplementary Fig. 7). In Tm-RNase H2 structure this region of DNA passes much closer to the protein and forms additional contacts with Phe83. In Ta-RNase H3 structure the region corresponding to the vicinity of Phe83 binds a sulfate ion through side chains of Asn154, Arg155, Asn184 and Arg185, which strongly suggests that this site would also bind the nucleic acid backbone, if a trajectory similar to the one observed in RNase H2 is possible. We attribute the difference in the trajectory of the non-cleaved strand to the different nature of the cleaved strand (RNA in RNase H3 structure and DNA with a single ribonucleotide in RNase H2 structure), which affects the overall geometry of the double helix. We therefore assume that for substrates with only one or several ribonucleotides in the cleaved strand Asn154, Arg155, Asn184 and Arg185 would bind a phosphate group downstream from the P-binding pocket.

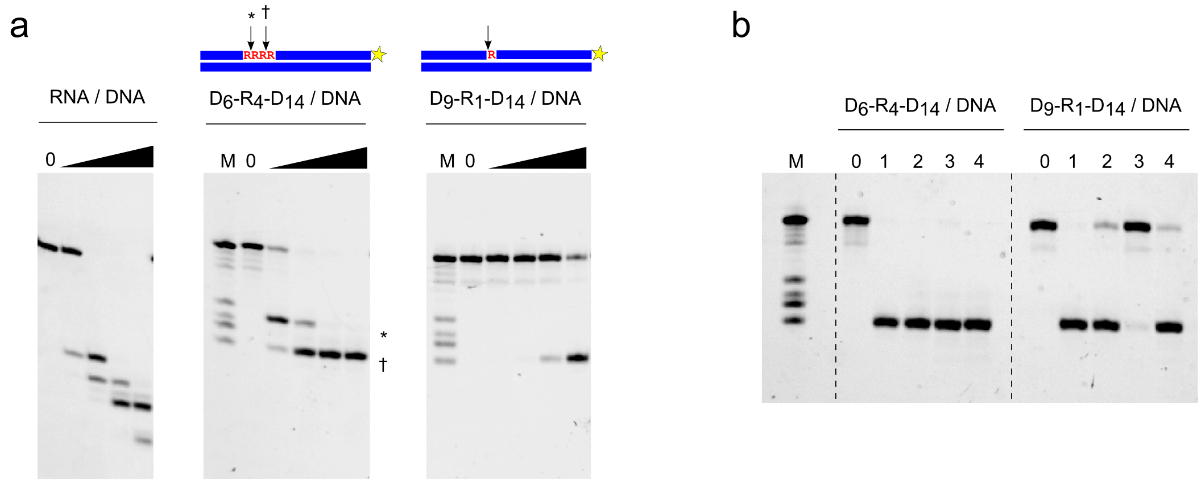

Supplementary Figure 1. Activity and substrate specificity of RNases H3. (a) Cleavage of model substrates by Ta-RNase H3. Each reaction contained 500 nM substrate. Lanes without the enzyme added are indicated by 0, and lanes marked with a triangle contain increasing amounts of protein (0.5, 5, 50, and 500 nM). The reactions were incubated at 37°C for 15 min in the presence of 5 mM $MgCl_2$. The cleavage products were analyzed on 20% Tris borate EDTA-urea polyacrylamide gels. The size of the products was estimated by comparisons with the marker indicated as M (products of alkaline hydrolysis of the cleaved strand). The schematic representation of the chimeric substrates shows DNA residues as blue and RNA residues as red. The observed cleavage sites are indicated with arrows, and the fluorescent label position is indicated with a yellow star. (b) Cleavage of model substrates by RNases H2 and H3. The lanes without enzyme added are indicated by 0. The reactions were incubated at 37°C for 15 min in the presence of 5 mM $MgCl_2$ and contained 500 nM *T. maritima* (Tm)-RNase H2 (lane 1), Ta-RNase H3 (lane 2), Tha-RNase H3 (lane 3), and Tl-RNase H3 (lane 4). The size of the products was estimated by comparisons with the marker indicated as M (products of alkaline hydrolysis of the cleaved strand).

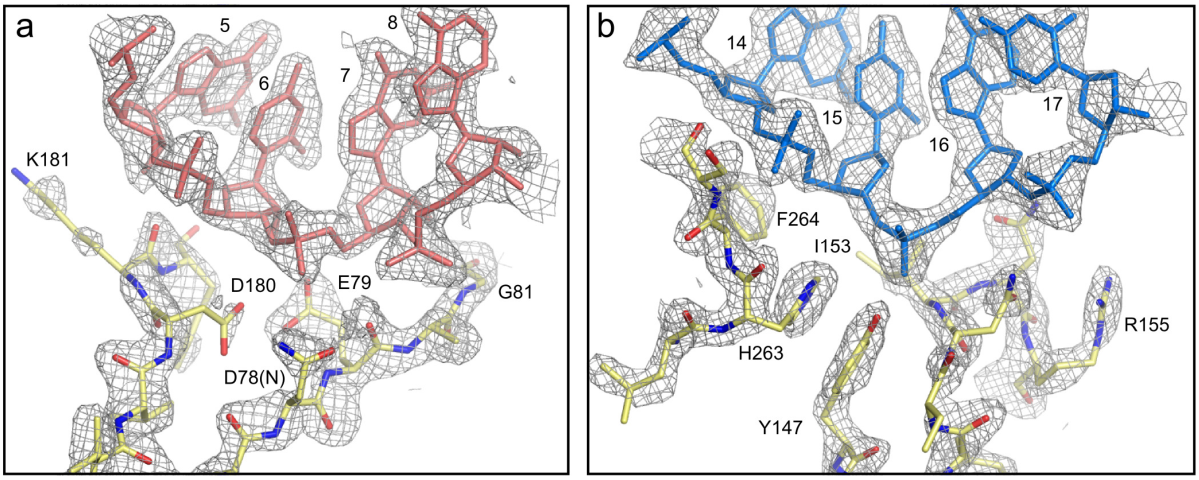

Supplementary Figure 2. Electron density maps. (a, b) Simulated annealing omit electron density maps contoured at 1.5 σ overlaid on the fragments of structure around the active site (a) and phosphate-binding pocket (b).

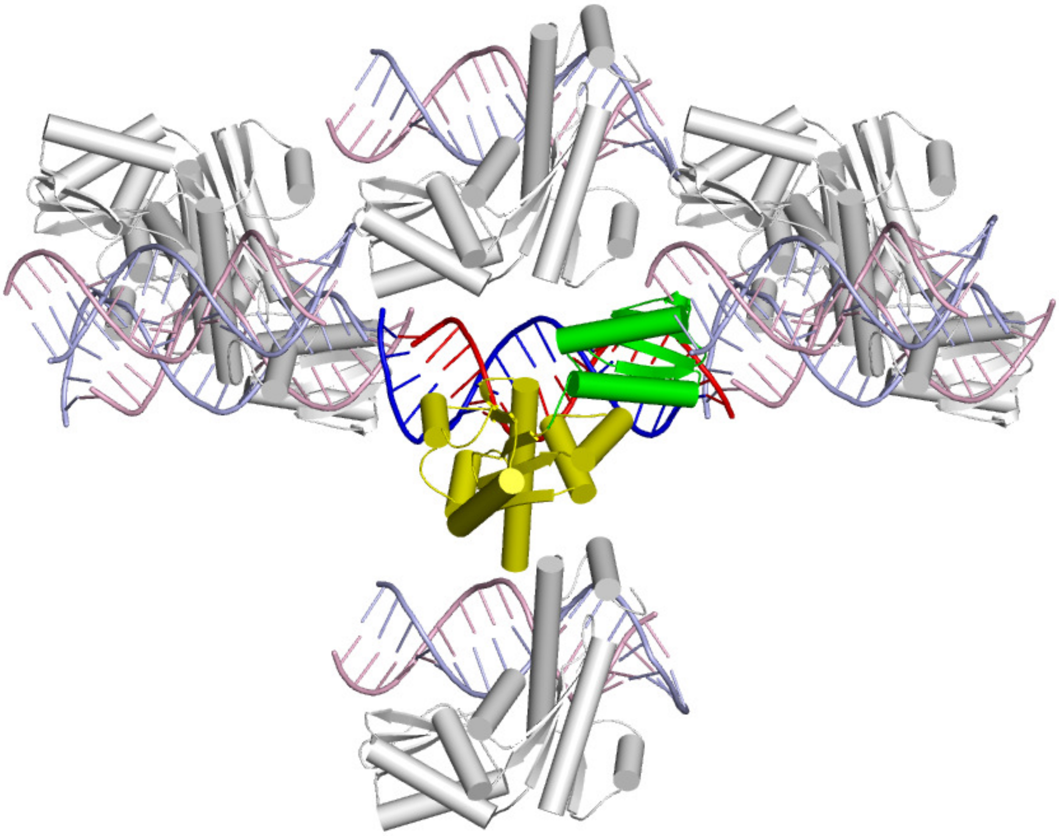

Supplementary Figure 3. Crystal packing interactions. One Ta-RNase H3 substrate complex molecule is colored as in Fig 1 and neighboring molecules in crystal lattice are in white for protein, light blue for DNA, and pink for RNA.

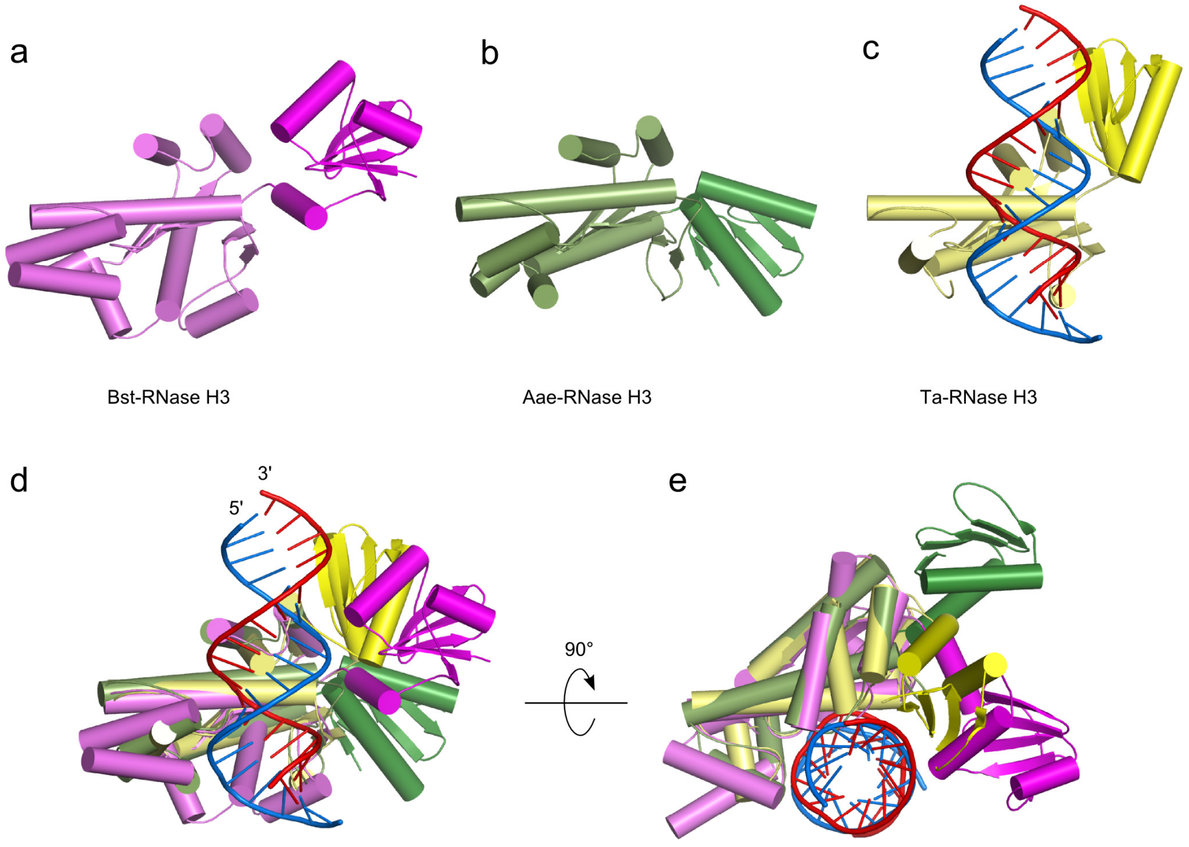

f

```
                                  *
Ta-RH3     1 ~~~~~MKLSSSEKEKLLKKLKALGAKEEKPPEHAQYRLRLNDAILTVYK~~~~SGSVVYGGKGREKLKELVAETVLSD~~~~~~~~~~~~~~~  69
Bst-RH3    1 MSNYVIQADQQLLDALRAHYE~~GALSDRLPAGALFAVKRPDVVITAYR~~~~SGKVLFQGKAAEQEAAKWISGASASNET~~~~~~~~~~~A  76
Aae-RH3    1 ~~MPSLKISPSEAEKIQNYLVSSGFRKINAPY~TLWALEGNGVKVYYYK~~~~TGSLLIQGKNSEKVLKEVLNLLE~~~~~~~~~~~~~~~~~  69
Tha-RH3    1 ~MNLTLKIPEELSEDLLSYLRGFGLK~EAWVRGAVWSLQGEGVKVSYFP~~~~SGVVLIQGKGAERIRDLLLEKLGG~~~~~~~~~~~~~~~~  71
Tl-RH3     1 ~~~~~~~MIDDLKNKLLKA~NLRIVSQKEIQYGIQFLIENCGYLRIYFRKNKSTTVDLSQIKPSCLEKIKHALGEEKPTFETLFCKDSDLSIQ  85
Tm-RH2       ~~~~~~~~~~~~~~~~~~~~~~~~~~~~~~~~~~~~~~~~~~~~~~~~~~~~~~~~~~~~~~~~~~~~~~~~~~~~~~~~~~~~~~~~~~~~~~

Ta-RH3    70 ~~~~~~~~~~~~TELPRIGCDEAGKGEFVGPLVVACIVADEKCLKRLIELGVKDSKKLSNEKVEELASEITETCHGKVKLLIPEKYNRAYSKF 150
Bst-RH3   77 DHQPSALAAHQLGSLSAIGSDEVGTGDYFGPIVVAAAYVDRPHIAKIAALGVKDSKQLNDEAIKRIAPAIMETVPHAVTVLDNPQYNRWQRSG 169
Aae-RH3   70 ~~~~~~~~~~~~~KKKLPGCDESGKGDIFGSLVLCCVCIPEENYLKVSSLNPRDTKRLSDKRVERLYLALKPLVKAYCYEIKPEEYNKLYRKF 149
Tha-RH3   72 ~~~~~~~~~~~~LEGPVVGCDESGKGDVFGPLVVCCAVVKPENYLKLVQVAPRDGKSLTPQQIAKKFSLMQDLVDIRCRIMPPEELNALYKDM 152
Tl-RH3    86 KRILENLPADPKNILPAIGSDEVGKGDVFGPVVVTAVYIGCKEYVALKDVDIKDSKSINDRKILEISKKIKNICPHAIVVFEPERLNEK~~~K 175
Tm-RH2     1 ~~~MGIDELYKKEFGIVAGVDEAGRGCLAGPVVAAAVVLEKEI~~~~~~EGINDSKQLSPAKRERLLDEIMEKAAVGIGIASPEEIDL~~~~~  79

Ta-RH3   151 KNINRLLEAVYREIVSDLCEKFS~~~PKVVVVDKFSNRAEEVLKDVV~~~~KGARLEVRPKAED~DLAVAAASIVAKAVRLKTMKELEKRF~~ 233
Bst-RH3  170 MPQTKMKALLHNRTLVKLVDAIAPAEPEAIIIDEFLKRDSYFRYLSDEDRIIRERVHCLPKAESVHVSVAAASIIARYVFLEEMEQLSRAV~~ 260
Aae-RH3  150 RNLNKMMTHFYKLLIERVKEECG~~~VSEVVVDKYQPSNPF~~~~~~~~~~~GEDVIFETEAER~NLAVAVASIFARYKFLQSLKEVEREL~~ 225
Tha-RH3  153 PNMNRVLTYLYKELLKEVPC~~~~~~QYPIVVDAYASRNPF~~~~~~~~~~~GPKVTFVPKGEK~NIAVACASIAARYHFLQWLQREG~~~~~ 222
Tl-RH3   176 INMNRLLEQMHIDAISRVLEKRS~~~SLIAVYDDFGAKKDRMRSV~~~~~~~EQLTLIGFKNGERNLAVAAASIVSRAEFLNWIETRSKFY~~ 256
Tm-RH2    80 YNIFNATKLAMNRALENLSVK~~~~~PSFVLVDGKGIELS~~~~~~~~~~~~~VPGTCLVKGDQKSKLIGAASIVAKVFRDRLMSEFHRMYPQ 154

Ta-RH3   234 ~KVKLPEGNT~GLAELLKKT~~~~PKELHEKLFKLHFSVGGKK~~~~~~~~~~~~~~~~~~~~~~~~~~~~~~~~~~~~~~~~~~~~~~~~~~ 270
Bst-RH3  261 ~GLLLPKGAGAIVDEAAARIIRARGEEMLETCAKLHFANTKKALAIAKRRK~~~~~~~~~~~~~~~~~~~~~~~~~~~~~~~~~~~~~~~~~~~ 310
Aae-RH3  226 ~GIKIPKGTSKEVKELAKSLK~~~~~~NPERFIKLNFNV~~~~~~~~~~~~~~~~~~~~~~~~~~~~~~~~~~~~~~~~~~~~~~~~~~~~~~ 257
Tha-RH3  223 ~~~~LPAGSSAKAMQEAHRILREDP~ERARRTIKLFFLQKE~~~~~~~~~~~~~~~~~~~~~~~~~~~~~~~~~~~~~~~~~~~~~~~~~~~~ 258
Tl-RH3   257 ~EMEIPLGASNKTIEFIRQFIKKYGLEELKKIAKLCFSNVRKFLTLEQNS~~~~~~~~~~~~~~~~~~~~~~~~~~~~~~~~~~~~~~~~~~~~ 305
Tm-RH2   155 FSFHKHKGYA~~~TKEHLNEIRKNGV~~~LPIHRLSFEPVLELLTDDLLREFFEKGLISENRFERILNLLGARKS~~~~~~~~~~~~~~~~~~ 223
```

(previous page) **Supplementary Figure 4. Comparison of Ta-RNase H3 with other RNases H3.** (a-c) Structure of Bst-RNase H3 (PDB ID:2D0A (21)) (a), Aae-RNase H3 (PDB ID: 3VN5 (22)) (b), and Ta-RNase H3 protein-substrate complex (c) in cartoon representation. N-domains are indicated with a darker shade of coloring. (d, e) Structures of Bst-RNase H3, Aae-RNase H3, and Ta-RNase H3 superimposed using the catalytic domain. (f) Sequence alignment of RNases H3 and RNase H2. Residues with an identity above 65% are shaded dark gray, and residues with a similarity above 65% are shaded light gray. Secondary structure elements are indicated above and below the alignment for Ta-RNase H3 and Tm-RNase H2, respectively, with helices shown as cylinders, and strands shown as arrows. Residues that interact with the nucleic acid in the crystal structures of protein-substrate complexes are colored red (interaction with RNA), blue (interaction with DNA), and purple (interaction with deoxynucleotides of the cleaved strand). Residues forming the phosphate-binding pocket are marked with asterisks. Ta, *T. ammonificans*; Bst, *B. stearothermophilus*; Aae, *A. aeolicus*; Tha, *T. albus*; Tl, *T. lettingae*; Tm, *T. maritima*.

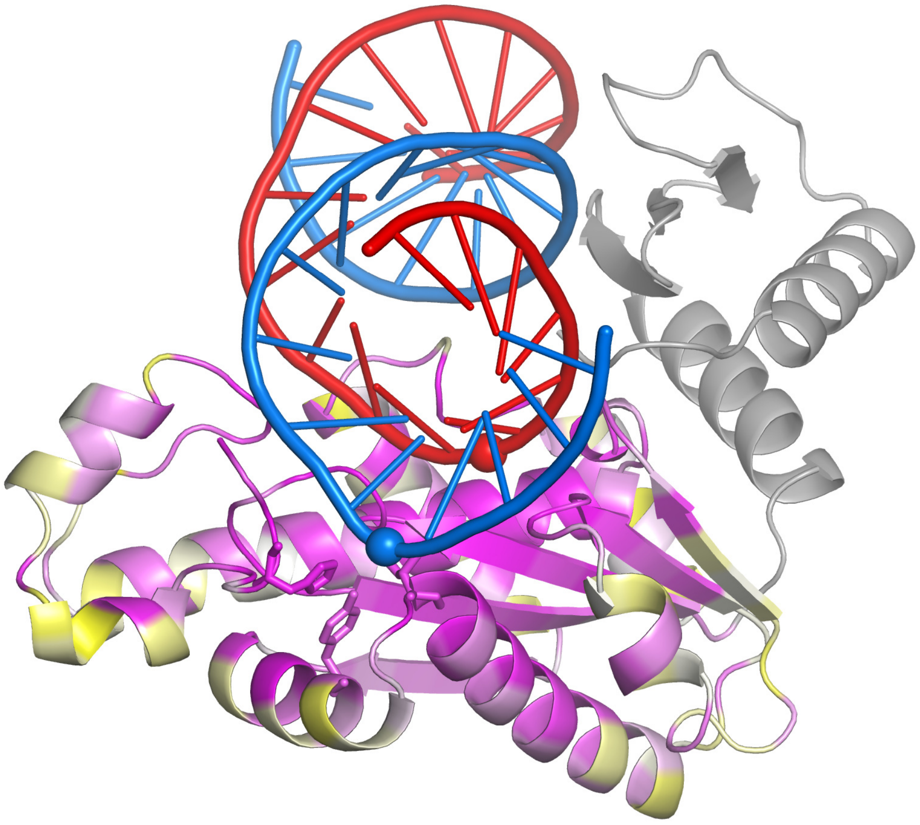

Supplementary Figure 5. Sequence conservation of the catalytic domain of Ta-RNase H3. Protein is shown in cartoon representation colored pink-white-yellow coding for high to low sequence conservation. The N-domain is shown in gray. RNA is shown in red and the DNA in blue. The residues forming the phosphate-binding pocket are shown as sticks and the bound phosphate group as a sphere. The coloring was calculated using ConSurf (33) based on an alignment of 199 RNase H3 sequences.

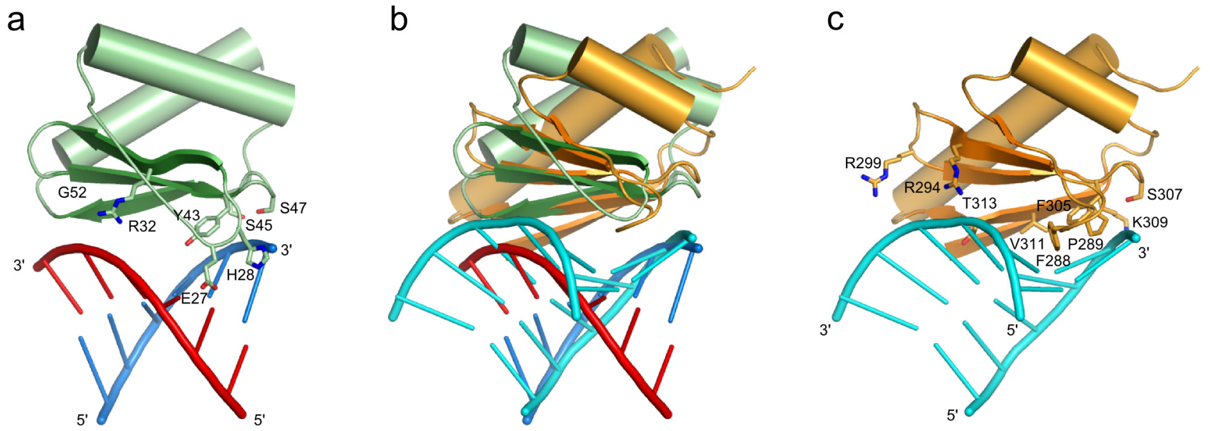

Supplementary Figure 6. Comparison of the substrate binding by the N-domain and TBP. (a) N-domain of RNase H3 bound to an RNA/DNA hybrid. DNA is shown in blue, and RNA is shown in red. Residues that interact with the nucleic acid are shown as sticks and labeled. (b) Superimposition of N-domain of RNase H3 and TBP domain of TATA-binding protein (PDB ID: 1NVP (23)) protein-nucleic acid complexes. (c) A single TBP domain of human TATA-binding protein bound to a dsDNA substrate. Residues that interact with the nucleic acid are shown as sticks and labeled.

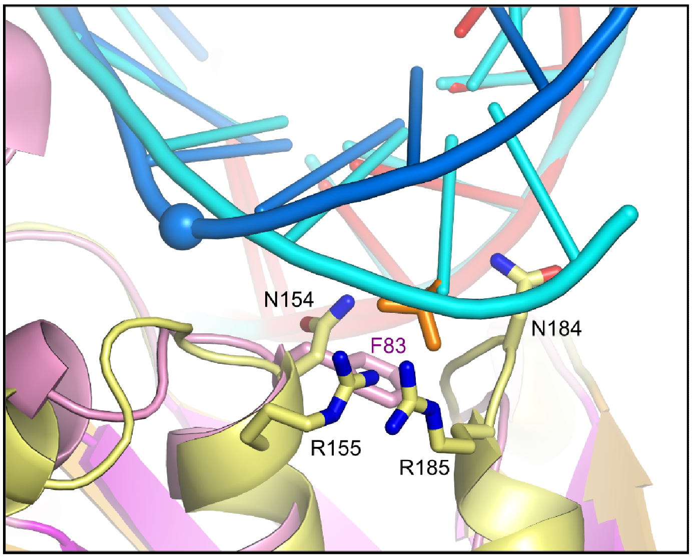

Supplementary Figure 7. Binding of the non-cleaved strand by RNase H2 (pink cartoon) and RNase H3 (yellow cartoon). The DNA in RNase H2 structure is shown in cyan and in RNase H3 structure in blue. The sulfate ion observed in Ta-RNase H3 structure is shown in orange and residues interacting with the ion in sticks.

SUPPLEMENTARY REFERENCE

33. Ashkenazy, H., Erez, E., Martz, E., Pupko, T. and Ben-Tal, N. (2010) ConSurf 2010: calculating evolutionary conservation in sequence and structure of proteins and nucleic acids. *Nucleic Acids Res*, **38**, W529-533.
